# Ultrafast Electronic Deactivation Dynamics of Xanthosine Monophosphate

**DOI:** 10.3390/molecules22010160

**Published:** 2017-01-18

**Authors:** Katharina Röttger, Rebecca Stellmacher, Mayra C. Stuhldreier, Friedrich Temps

**Affiliations:** 1 Institute of Physical Chemistry, Christian-Albrechts-University Kiel, Olshausenstr. 40, 24098 Kiel, Germany; stellmacher@phc.uni-kiel.de (R.S.); mayra.stuhldreier@gmx.de (M.C.S.); 2 Centre for Process Innovation, Wilton Centre, Wilton, Redcar TS10 4RF, UK

**Keywords:** biophysics, DNA, RNA, ultrafast photochemistry, xanthosine monophosphate, XMP, transient absorption, fluorescence up-conversion

## Abstract

Ultrafast energy dissipation is a crucial factor for the photostability of DNA and RNA, but even some of the key electronic deactivation pathways in monomeric nucleic acid building stones are still controversial. Here, we report on the excited-state dynamics of the rare nucleotide xanthosine monophosphate as a function of deprotonation state (XMP vs. XMP${}^{-}$) and excitation wavelength ($\lambda_{\mathrm{pump}}=$ 278–243 nm) by femtosecond time-resolved fluorescence and absorption spectroscopy. We show that the predominating relaxation channel leads to a return of the photo-excited molecules to the electronic ground state in *τ*∼1 ps. The mechanism likely involves an out-of-plane deformation of the five-membered ring, different from the main electronic deactivation pathways in the canonical purine bases adenine and guanine. The results are discussed in terms of the structural and electronic differences of XMP compared to the canonical nucleotides.

## 1. Introduction

**Figure 1:**
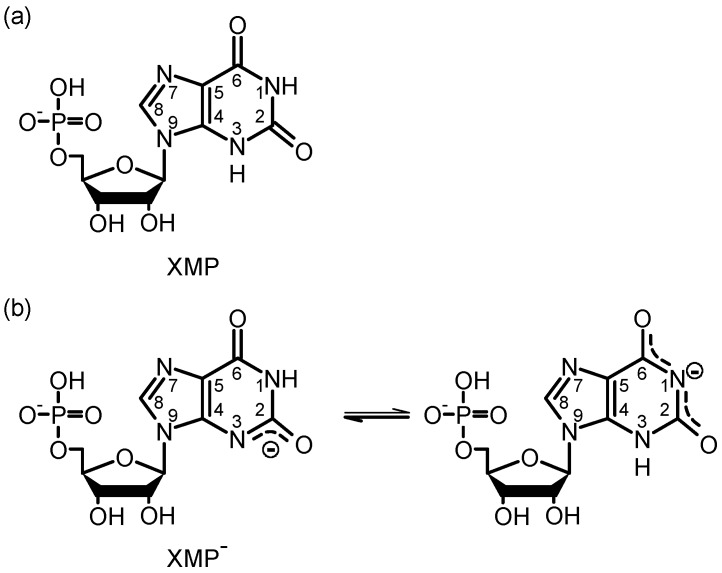
Structures of xanthosine monophosphate at (**a**) acidic and (**b**) neutral pH. The numbers 1–9 indicate the atom labels in the purine ring.

The 6-oxopurine xanthine, its nucleoside xanthosine and its nucleotide xanthosine monophosphate (XMP) play important functions as intermediates in various metabolic pathways, for example in the oxidative deamination of guanine, the formation of guanosine monophosphate (GMP) via XMP, the biosynthesis of caffeine by methylation of xanthosine and as a rare base in ribonucleic acids (RNAs) [1,2]. Xanthine differs from guanine only by a carbonyl group at the C(2) position instead of the amino group and therefore possesses no double bond at the C(2)-N(3) position (see Figure 1). This leads to remarkable changes in the electronic structure. The most obvious one is the extraordinary increase in acidity (p*K*${}_{a}$≈ 5.7) compared to all common other purine bases (e.g., p*K*${}_{a}$≈ 9.2 for guanosine), which has been shown in investigations of the gas and solution phase structures [3,4,5,6,7,8,9] and in experimental [10,11,12,13] and theoretical [14] investigations of its electronic properties.

Whereas xanthine in its ground state has been extensively studied, the available information on the excited electronic state is very limited. The photophysics of the two thermodynamically most stable tautomers, the canonical N(9)H and the N(7)H diketo tautomers, have recently been investigated theoretically by Yamazaki et al. [15]. Two electronic relaxation mechanisms were proposed: a conical intersection (CI) between the first ${}^{1}\pi \pi^{*}$ excited state and the ground state that is accessible via an out-of-plane deformation of the five-membered ring can lead to an efficient direct re-population of the electronic ground state. This relaxation pathway was predicted to be more likely for the N(7)H tautomer; the N(9)H tautomer was supposed to exhibit a small potential energy barrier en route to this CI. The second pathway back to the electronic ground state was proposed to proceed via NH dissociation in the five-membered ring. That mechanism was suggested to be more likely for the N(9)H tautomer. For the nucleoside and nucleotide, however, the NH dissociation channel is blocked by the sugar moiety. The photo-induced radiationless dynamics of the N(9) nucleoside and nucleotide should therefore be controlled by the puckering motion of the five-membered ring as well. This makes an important difference to other purine bases like guanine and adenine, where electronic deactivation involves ethylenic out-of-plane deformation of the six-membered ring at the C2-N3 position [16,17,18,19,20,21]. Experimentally, the electronic deactivation dynamics has been investigated to date only for several methylated xanthine derivatives by Chen et al. [22], who used transient absorption spectroscopy at probe wavelengths of 570 and 250 nm and found the out-of-plane deactivation of the five-membered ring to be a plausible relaxation pathway.

In this work, we focus on the photodynamics of the nucleotide XMP for two reasons: First, unlike xanthine and its methyl derivatives, XMP is soluble in water, allowing us to perform time-resolved fluorescence and absorption spectroscopy on the molecules in aqueous solution at room temperature at different pH values to explore the effect of the neutral and anionic states of the xanthosine moiety. Second, the number of possible tautomers of the deprotonated form of XMP is reduced to the N(3)H-N(9)R and N(1)H-N(9)R diketo forms (cf. Figure 1) by the ribosyl group at N(9). The structural properties and tautomeric equilibria of XMP in aqueous solution are well known by the investigation of the micro-acidity constants of the N(3)H and N(1)H groups by Massoud et al. [7]. Additionally, the structure has been studied by resonance Raman spectroscopy by Gogia et al. [9]. The results of both groups revealed that XMP is deprotonated at physiological pH values in the xanthine moiety, preferentially at the N(3) position. Gogia et al. showed that the deprotonation leads to a weakening of both carbonyl bonds, especially at the C(2)O position, due to electron delocalization following the proton loss. In the free, neutral purine base xanthine, on the other hand, a considerable amount of conjugation was found between the NH and the carbonyl groups of the six-membered ring, as was shown by X-ray photoemission and near-edge X-ray absorption fine structure (NEXAFS) spectroscopy [13]. The electronic structures of XMP with neutral xanthine and XMP with anionic xanthine may therefore be quite similar regarding the degree of electron delocalization. XMP with neutral xanthine can exist preferentially only in slightly acidic solution (pH <5) [6,7]. In the following, we will refer to the nucleotide with neutral xanthine as XMP and with deprotonated xanthine as XMP${}^{-}$.

## 2. Results

### 2.1. Static Absorption and Fluorescence Spectra

**Figure 2:**
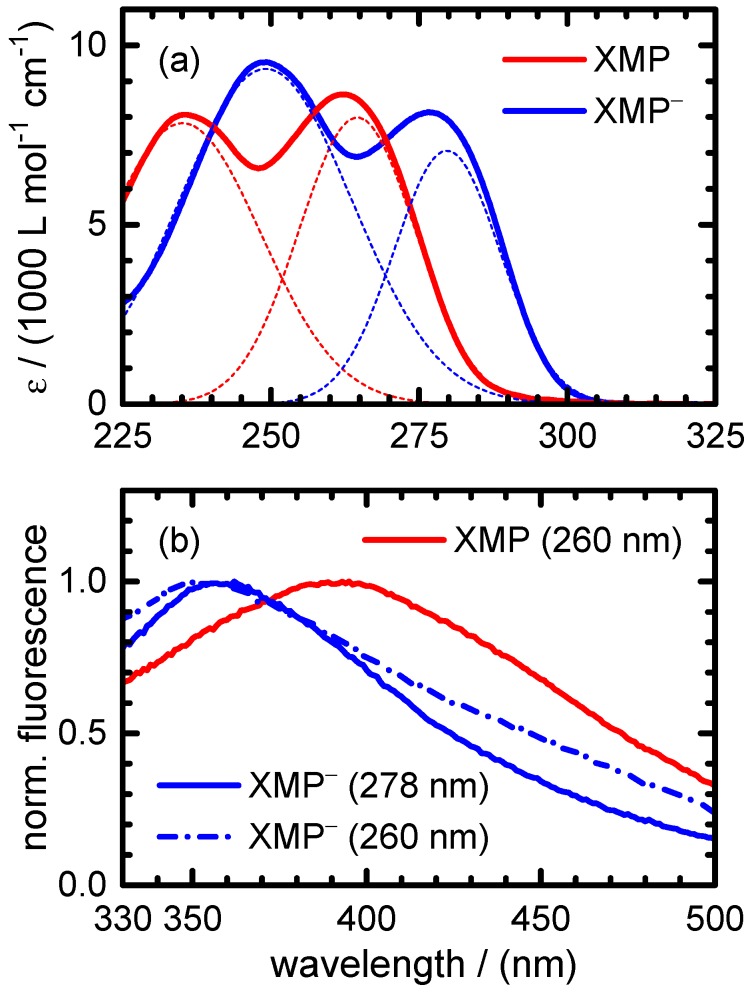
(**a**) Static UV absorption and (**b**) fluorescence spectra of XMP (red) and XMP${}^{-}$ (blue). The thin dotted lines in (**a**) show the contributions to the absorption spectra by the S${}_{1}$ and S${}_{3}$ bands; the wavelengths in the labels in (**b**) denote the respective pump wavelengths used to record the spectra.

The static absorption and fluorescence spectra of XMP and XMP${}^{-}$ are depicted in Figure 2. As has been discussed by Cavalieri et al. [3] and Lichtenberg et al. [4], both species exhibit two strong absorption bands in the near UV, which are attributed to two close-lying ${}^{1}\pi \pi^{*}$ transitions, as in other purine bases. Based on the ab initio calculations of Yamazaki et al., the ${}^{1}\pi \pi^{*}$ states of the N(9)H diketo tautomer of xanthine should be labelled S${}_{1}$, resp. S${}_{3}$ at vertical excitation. The S${}_{2}$ state has a ${}^{1}n\pi^{*}$ character and a very low oscillator strength, i.e., is optically dark in the absorption spectrum [15]. The spectral characteristics of both ${}^{1}\pi \pi^{*}$ absorption bands are summarized in Table 1.

Compared to XMP, the ${}^{1}\pi \pi^{*}$ transitions of XMP${}^{-}$ are red-shifted by ∼14 nm, and the second ${}^{1}\pi \pi^{*}$ transition gains intensity at the expense of the first. The fluorescence spectra of both species feature a broad and unstructured emission with maxima at 390 nm (XMP) and 355 nm (XMP${}^{-}$). Interestingly, the fluorescence maximum for XMP${}^{-}$ is blue-shifted compared to XMP, which contrasts with the shift of the absorption bands. This indicates a considerably larger energy difference in the Franck–Condon (FC) window to the S${}_{0}$ state. While the fluorescence spectra of XMP show no dependence on excitation wavelength, the spectrum of XMP${}^{-}$ after excitation to the second ${}^{1}\pi \pi^{*}$ state exhibits increased emission at visible wavelengths. This may be due to a fluorescing state with a low potential energy barrier along the relaxation pathway on the excited electronic potential energy hypersurface (PEHS).

### 2.2. Time-Resolved Experiments

#### 2.2.1. XMP

For the time-resolved experiments on XMP, two excitation wavelengths were selected, $\lambda_{\mathrm{pump}}=260$ nm (fluorescence and transient absorption measurements) and $\lambda_{\mathrm{pump}}=243$ nm (transient absorption measurements only). Inspecting the UV absorption spectra in Figure 2, the first and second ${}^{1}\pi \pi^{*}$ states are populated at both wavelengths with relative yields of ∼84% and 16% ($\lambda_{\mathrm{pump}}=260$ nm) and ∼10% and 90% ($\lambda_{\mathrm{pump}}=243$ nm), respectively.

**Figure 3:**
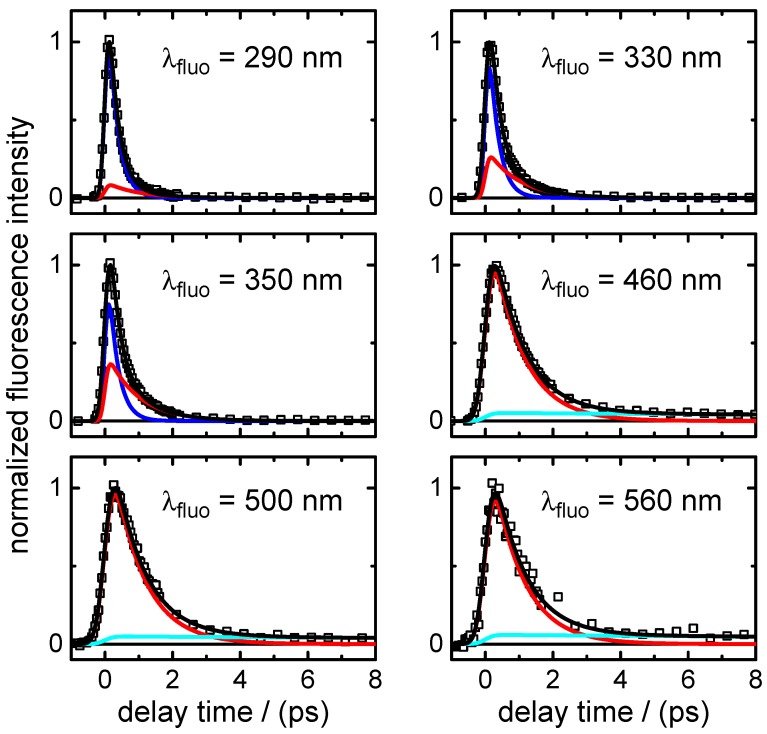
Fluorescence-time profiles of XMP after excitation at $\lambda_{\mathrm{pump}}=260$ nm at six selected emission wavelengths in the range $\lambda_{\mathrm{fluo}}$ = 290–560 nm. Open squares represent data points and black lines the overall fit functions. The single contributions are shown in blue ($\tau_{1,\mathrm{fluo}}$), red ($\tau_{2,\mathrm{fluo}}$) and cyan ($\tau_{3,\mathrm{fluo}}$). For clarity, the data points are displayed in the different panels only up to Δt=8 ps, but were recorded in the experiment and analysed up to a delay time of Δt=60 ps.

**Figure 4:**
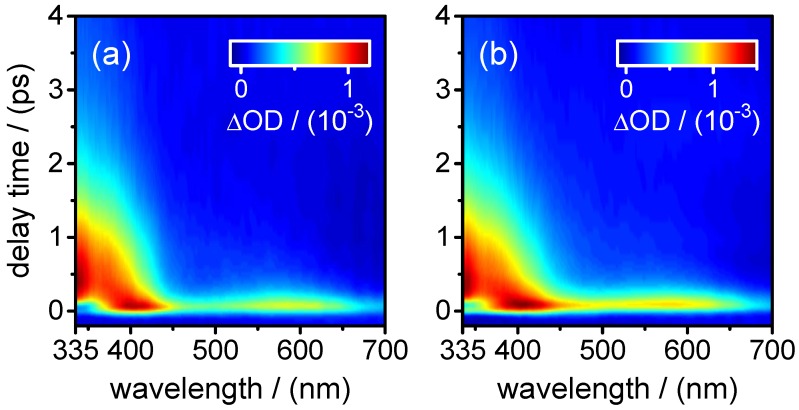
Two-dimensional maps of the observed changes in optical density in the transient absorption experiments on XMP after excitation at: (**a**) $\lambda_{\mathrm{pump}}=260$ nm; and (**b**) $\lambda_{\mathrm{pump}}=243$ nm.

**Figure 5:**
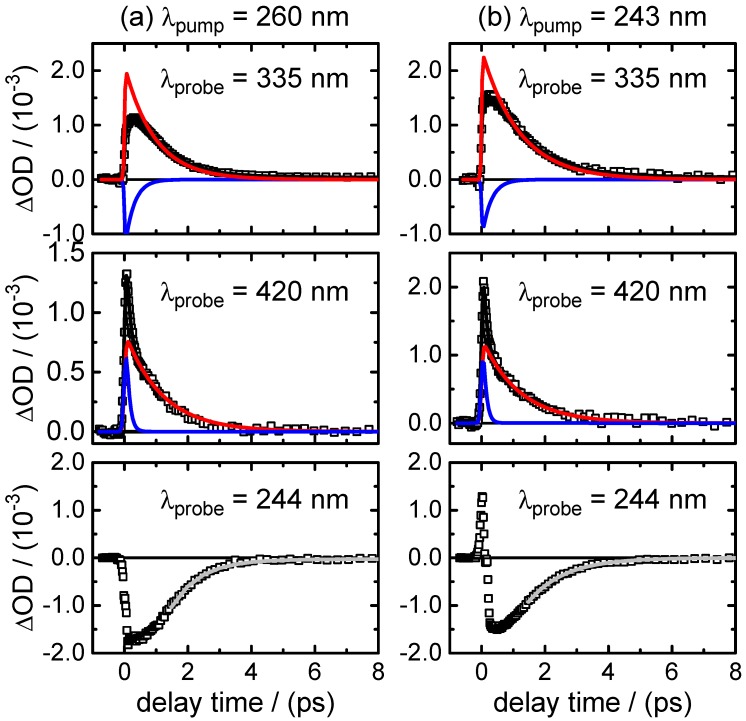
Time profiles of the change in optical density after excitation of XMP at (**a**) $\lambda_{\mathrm{pump}}=260$ nm and (**b**) $\lambda_{\mathrm{pump}}=243$ nm. Open squares represent data points and black lines the overall fit functions. The single contributions in the recorded traces at 335 and 420 nm are shown in blue ($\tau_{1}$) and red ($\tau_{2}$). The initial spike in the time profile with 243 nm pump/244 nm probe showing the recovery of the ground state bleach (GSB) signal is attributed to residual cross phase modulation (XPM). The light grey lines in the bottom panels ($\lambda_{\mathrm{probe}}=244$ nm) show asymptotic single exponential fits to the observed ground state recovery (GSR) time profiles beginning at Δt=1.75 ps after $t_{0}$ (see the text).

**Figure 6:**
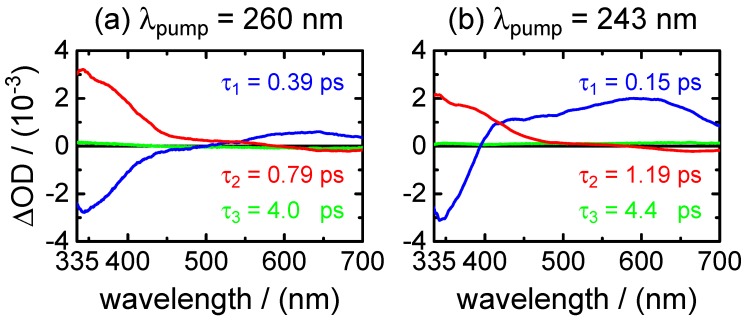
Decay time-associated difference spectra (DADS) vs. probe wavelength for XMP after excitation at (**a**) $\lambda_{\mathrm{pump}}=260$ nm and (**b**) $\lambda_{\mathrm{pump}}=243$ nm. $\tau_{1}$: blue; $\tau_{2}$: red; $\tau_{3}$: green.

(i) Transient fluorescence: Fluorescence lifetimes were measured after excitation at $\lambda_{\mathrm{pump}}=260$ nm at six emission wavelengths, from $\lambda_{\mathrm{fluo}}=290$ nm to $\lambda_{\mathrm{fluo}}=560$ nm, to gain insight into the dynamics of the optically bright photo-excited state(s). The recorded time traces are shown in Figure 3. A simultaneous nonlinear least squares fit analysis of the data yielded two dominating time constants,
  $$\begin{array}{cc} \tau_{1,\mathrm{fluo}} & =0.28\pm 0.01\;\;\mathrm{ps},\\ \tau_{2,\mathrm{fluo}} & =0.91\pm 0.01\;\;\mathrm{ps}. \end{array}$$
  The fast component ($\tau_{1,\mathrm{fluo}}$) was found only at the shortest fluorescence wavelengths ($\lambda_{\mathrm{fluo}}\leq 350$ nm) and resembles the decay time in the same spectral range of the stimulated emission contribution to the transient absorption maps (see below). The component decaying with $\tau_{2,\mathrm{fluo}}$ was the main one at all fluorescence wavelengths ≥460 nm. Additionally, a very small third exponential with an amplitude of 3%–4% decaying with $\tau_{3,\mathrm{fluo}}=36\pm 8$ ps was required for the fitting to the data points at longer delay times (Δt≤60 ps) at wavelengths of $\lambda_{\mathrm{fluo}}\geq 460$ nm. A summary of the time-resolved fluorescence results for XMP is given in Table A1.
(ii) Broadband transient absorption: The two-dimensional spectro-temporal absorption maps for XMP showing the change in optical density observed by the broadband transient absorption experiments after excitation at $\lambda_{\mathrm{pump}}=260$ nm and $\lambda_{\mathrm{pump}}=243$ nm are shown in Figure 4. Both absorption maps are similar and feature three overlapping positive bands with maxima at $\lambda_{\mathrm{probe}}\leq 340$ nm, $\lambda_{\mathrm{probe}}$∼420 nm and $\lambda_{\mathrm{probe}}$∼550–600 nm. The positive contributions are attributed to excited-state absorption (ESA). In the near-UV probe range, a delayed rise of the positive band is observed, which can at least partially be explained by an overlapping negative contribution by stimulated emission (SE). Both the spectral region and the decay time (see below) of the negative contribution are consistent with the fluorescence up-conversion measurements above. The positive band at 420 nm seems to feature a blue-shift of the maximum within the first 500 fs, most likely due to the SE contribution at UV wavelengths and the faster decay of the absorption band in the UV.
  For a first orientation on the ensuing dynamics, three to four time profiles were chosen in each of the above ESA bands and modelled by simultaneous least squares fitting using a sum of exponentials. Exemplary time profiles for $\lambda_{\mathrm{probe}}=335$ nm and 420 nm together with the fitted curves are depicted in Figure 5 (see Table A2 for the obtained fit parameters). These preliminary results were then completed by global analyses of the two-dimensional spectro-temporal absorption maps in Figure 4 using the method of singular value decomposition (SVD [23,24,25]; see Section 4).
  The resulting time constants are listed in Table 2; the related decay time-associated difference spectra (DADS) are shown in Figure 6. As can be seen, three decay times were needed for satisfactory modelling of the data. Taking the average values at the two excitation wavelengths, the results are:
  $$\begin{array}{cc} \tau_{1} & =0.27\pm 0.15\;\;\mathrm{ps},\\ \tau_{2} & =1.00\pm 0.20\;\;\mathrm{ps},\\ \tau_{3} & =4.20\pm 1.20\;\;\mathrm{ps}. \end{array}$$
(iii) Ground-state recovery: The ground state recovery (GSR) resulting from the ultrafast radiationless electronic deactivation process was probed by deep-UV single-colour measurements at $\lambda_{\mathrm{probe}}=244$ nm. The observed time profiles showing the ground state bleach (GSB) signal are given in Figure 5 (see the bottom row). The data could by nicely modelled by the difference of two exponentials, as required for the expected GSR kinetics. The resulting fit parameters can be found in Table A2. To escape from the strong correlation between the fitted values and their large error margins, we also modelled the GSR time profiles asymptotically by fitting a single exponential to describe the approach of the signal to its final ΔOD=0 value, which gave a value of τ=1.1±0.1 ps. However, a more realistic measure for the GSR time may be obtained by taking the time value needed to reach the 90% recovery level. From Figure 5, this can be seen to be of the order of:
  $$\begin{array}{cc} \tau_{\mathrm{GSR}} & ∼3.5\;\;\mathrm{ps}. \end{array}$$
  A longer-lived excited-state contribution as hinted at by the fluorescence up-conversion measurements was not supported by the ESA or GSR time profiles.

#### 2.2.2. XMP${}^{-}$

The dynamics of XMP${}^{-}$ in the phosphate buffered solution at pH 7 were studied after excitation at three selected pump wavelengths, $\lambda_{\mathrm{pump}}=278$ nm (transient absorption measurements), $\lambda_{\mathrm{pump}}=260$ nm (transient fluorescence and transient absorption measurements) and $\lambda_{\mathrm{pump}}=243$ nm (transient absorption measurements only). Inspecting the static absorption spectra in Figure 2, the first and second ${}^{1}\pi \pi^{*}$ states are populated with fractions of 84% and 16% ($\lambda_{\mathrm{pump}}=278$ nm), 8% and 92% ($\lambda_{\mathrm{pump}}=260$ nm) and <1% and >99% ($\lambda_{\mathrm{pump}}=243$ nm), respectively.

**Figure 7:**
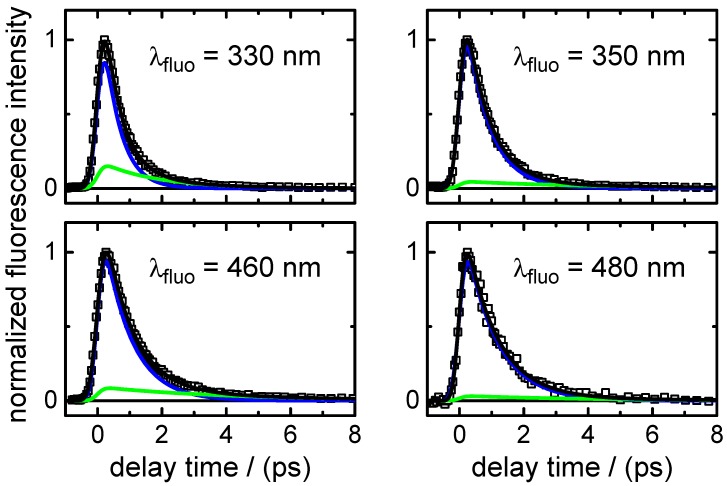
Fluorescence time profiles of XMP${}^{-}$ after excitation at $\lambda_{\mathrm{pump}}=260$ nm measured at four selected emission wavelengths in the range $\lambda_{\mathrm{fluo}}$ = 330–480 nm. Open squares represent data points, black lines the overall fit functions. The single contributions are shown in blue ($\tau_{1,\mathrm{fluo}}$) and green ($\tau_{2,\mathrm{fluo}}$).

**Figure 8:**
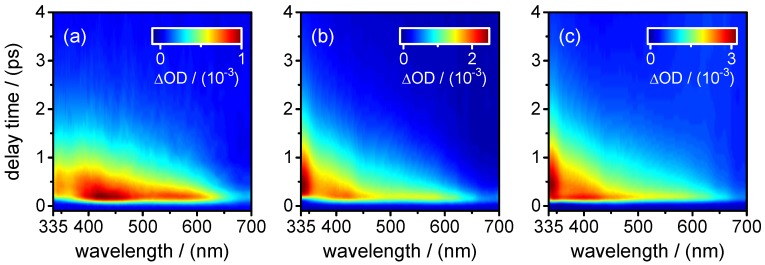
Two-dimensional maps of the observed changes in optical density in the transient absorption experiments on XMP${}^{-}$ after excitation at: (**a**) $\lambda_{\mathrm{pump}}=278$ nm; (**b**) $\lambda_{\mathrm{pump}}=260$ nm; and (**c**) $\lambda_{\mathrm{pump}}=243$ nm.

**Figure 9:**
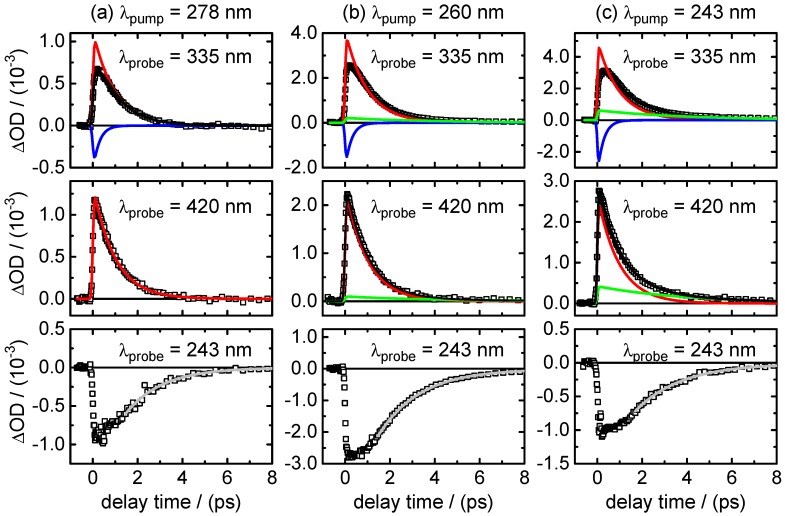
Time profiles of the change in optical density after excitation of XMP${}^{-}$ at: (**a**) $\lambda_{\mathrm{pump}}=278$ nm; (**b**) $\lambda_{\mathrm{pump}}=260$ nm; and (**c**) $\lambda_{\mathrm{pump}}=243$ nm. Open squares represent the data points and black lines the overall fit function. The single contributions are shown in blue ($\tau_{1}$), red ($\tau_{2}$) and green ($\tau_{3}$). The light grey lines in the bottom panels ($\lambda_{\mathrm{probe}}=243$ nm) show asymptotic single exponential fits to the observed GSR time profiles beginning at Δt=1.75 ps after $t_{0}$.

**Figure 10:**
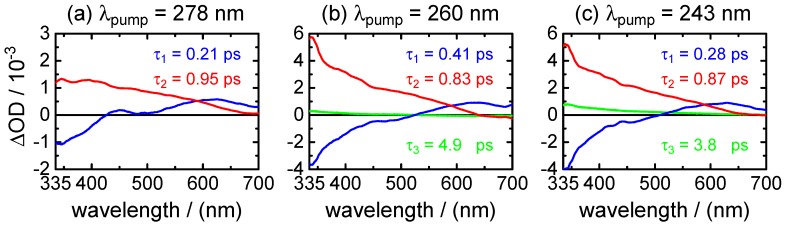
Decay time-associated difference spectra (DADS) vs. probe wavelength for XMP${}^{-}$ after excitation at: (**a**) $\lambda_{\mathrm{pump}}=278$ nm; (**b**) $\lambda_{\mathrm{pump}}=260$ nm; and (**c**) $\lambda_{\mathrm{pump}}=243$ nm. $\tau_{1}$: blue; $\tau_{2}$: red; $\tau_{3}$: green.

(i) Transient fluorescence: The fluorescence lifetime of XMP${}^{-}$ was measured following excitation at $\lambda_{\mathrm{pump}}=260$ nm at four wavelengths, from $\lambda_{\mathrm{fluo}}=330$ nm to $\lambda_{\mathrm{fluo}}=480$ nm. The recorded time profiles are depicted in Figure 7. Interestingly, the time profiles could not be modelled using two exponentials with varying amplitudes and fixed lifetime values, as had been possible for XMP. Instead, the decay rates slow down gradually with increasing fluorescence wavelengths (see Section 3). The major fluorescence lifetime component (see Table A3 for the list of fit parameters) increase in the range:
  $$\begin{array}{cc} \tau_{1,\mathrm{fluo}} & =(0.51\pm 0.01)\;\mathrm{ps}\;\mathrm{to}\;(1.0\pm 0.2)\;\mathrm{ps}. \end{array}$$
  Adding a minor second component between $\tau_{2,\mathrm{fluo}}=\left(1.7\pm 0.5\right)\;\mathrm{ps}\;\mathrm{to}\;\left(4.0\pm 1.0\right)$ ps improved the fits at long delay times, but large error margins owing to low amplitudes (∼11%–3%) make this component less reliable.
(ii) Broadband transient absorption: The recorded transient absorption maps for XMP${}^{-}$ are displayed in Figure 8. The data resemble those for XMP, with three similar overlapping ESA regions, but the shapes and positions of the ESA band maxima shift slightly from XMP to XMP${}^{-}$. Moreover, they seem to depend on the excited ${}^{1}\pi \pi^{*}$ state. After excitation to the first ${}^{1}\pi \pi^{*}$ state at $\lambda_{\mathrm{pump}}=278$ nm, the ESA maxima are at $\lambda_{\mathrm{probe}}<340$ nm, ∼440 nm and ∼600 nm (Figure 8a). On excitation to the second ${}^{1}\pi \pi^{*}$ state at $\lambda_{\mathrm{pump}}=260$ nm and 243 nm, the ESA bands shift to the blue by ∼20 nm, and the ESA band in the UV gains in intensity (Figure 8b,c). Consistent with the fluorescence up-conversion data above, there is a negative contribution by SE that leads to an apparently delayed rise of the ESA in the UV.
  Preliminary least squares fits to the transient absorption data at exemplary probe wavelengths are illustrated in Figure 9 (cf. Table A4 for the respective parameters). The preliminary fits were subsequently replaced, as before for XMP, by singular value analyses. The resulting global time constants are compiled in Table 3, and the corresponding DADS are displayed in Figure 10. Averaging the time constants at the three pump wavelengths, the results are:
  $$\begin{array}{cc} \tau_{1} & =0.30\pm 0.11\;\;\mathrm{ps},\\ \tau_{2} & =0.88\pm 0.08\;\;\mathrm{ps},\\ \tau_{3} & =4.30\pm 1.00\;\;\mathrm{ps}. \end{array}$$
(iii) Ground-state recovery: The recovery of the population in the electronic ground state resulting from the observed ultrafast non-radiative electronic deactivation is illustrated by the time profiles at $\lambda_{\mathrm{probe}}=243$ nm in Figure 9. The best fit parameters for these data can be found in Table A4. As before for XMP, however, the precise values need to be viewed with caution due to the strong correlation between the positive and negative exponentials. Fitting asymptotic single exponentials to the GSR time profiles to describe the approach to their final ΔOD=0 values produced time constants around τ=1.7±0.5 ps. More realistically, Figure 9 gives GSR times referenced to the 90% recovery level of:
  $$\begin{array}{cc} \tau_{\mathrm{GSR}} & ∼4.4-5.9\;\;\mathrm{ps}, \end{array}$$
  increasing slightly with the excitation wavelength.

## 3. Discussion

### 3.1. XMP

The time-resolved experiments on neutral XMP in aqueous solution under acidic conditions demonstrate an ultrafast radiationless deactivation of the photo-excited ${}^{1}\pi \pi^{*}$ electronic state(s) to the ground state that is characterized by two time constants, $\tau_{1}=0.27$ ps and $\tau_{2}=1.0$ ps. Both values have to be associated with different processes.

Several arguments let us assign the sub-picosecond lifetime $\tau_{1}$ to the decay dynamics of the initial FC excited state. First, $\tau_{1}$ is the predominating decay time of the fluorescence at wavelengths in the UV close to the excitation wavelengths. Second, as shown by the DADS in Figure 6 and by the selected time profiles in Figure 5, $\tau_{1}$ also describes the decay of the SE contribution (negative) in the transient absorption maps observed at $\lambda_{\mathrm{probe}}<$ 400–450 nm. At the same time, it is associated with the fast decay component of the (positive) fast ESA signal at $\lambda_{\mathrm{probe}}>$ 400–450 nm. The positive (ESA) amplitude increases towards longer probe wavelengths at the expense of the negative (SE) amplitude in the near-UV. The observation that the SE and fast ESA contributions could not be separated by the SVD analysis, but are described by a single DADS component, with a common single time constant ($\tau_{1}$), strongly supports that they are related to the initial FC excited state.

The DADS component corresponding to time constant $\tau_{2}=$ 0.8–1.2 ps, on the other hand, remains positive in the entire probe wavelength range. Its observation hints at an ESA contribution from a “relaxed” excited state. The most plausible assignment for $\tau_{2}$ is to the transition through the CI that connects the first ${}^{1}\pi \pi^{*}$ state to the ground state. Its short value indicates a rapid, barrierless wave packet motion in the direction of the CI. A definitive assignment would require QM/MM dynamics simulations of the first three electronically-excited states, as were recently reported for purine and 9-methylpurine [26]. However, the single-colour experiments at $\lambda_{\mathrm{probe}}=244$ nm confirmed that the initially excited molecules return to the electronic ground state within $\tau_{\mathrm{GSR}}$∼3.5 ps. This value has to be slightly longer than $\tau_{2}$ because it includes the vibrational cooling time of the initially very “hot” molecules after their return through the CI to the S${}_{0}$ ground state. The vibrational cooling is likely described by the third decay component $\tau_{3}$∼4.2 ps, which has a DADS with a very low amplitude. Eventually, we note that the absence of sizeable differences after selective photoexcitation to either the first or the second ${}^{1}\pi \pi^{*}$ state hints at an ultrafast internal conversion from S${}_{3}$ to S${}_{1}$ faster than our experimental time resolution. The longer-lived fluorescence component decaying with $\tau_{3}$∼36 ps suggested by the up-conversion data was barely above the detection experimental limit, such that it is hard to judge its reliability and origin. A slightly increased scattered light level in the measurement cannot be fully ruled out. A trapping of a small fraction of the excited wave packet in a shallow potential energy well along some different deactivation pathway could give a plausible explanation for the longer-lived component. Another possibility is a transfer of a fraction of the excited-state population to the ${}^{1}$n$\pi^{*}$ state [22]. In this case, the lack of a corresponding excited-state absorption signal could be due to a very weak ${}^{1}$n$\pi^{*}\to$ S${}_{n}$ oscillator strength. We note, meanwhile, that no comparable long-lived decay component was needed for modelling the ground state recovery data.

Yamazaki et al. proposed the coordinate connecting the first ${}^{1}\pi \pi^{*}$ excited state with the electronic ground state to be an out-of-plane deformation of the five-membered ring [15]. This relaxation pathway differs compared to the usual situation in the purine bases, where relaxation is supposed to occur via an ethylenic puckering motion of the six-membered ring. This usual pathway is not available in XMP, as there is no double bond at C2-N3. Given the observed ∼1 ps fluorescence and ESA decay time constants, a slight potential energy barrier, as proposed by the calculations of Yamazaki et al., seems to be highly unlikely at least for XMP in water. This might be a result of the replacement of the H atom at the N(9) position by the ribosyl-phosphate group. Although sugar-phosphate groups do not usually have large impacts on the photodynamics of other purine bases, it may be of larger importance here, where the five-membered ring is involved. A comparative study of xanthine and its nucleoside xanthosine would help to shed light on this question, but is difficult regarding the low solubility of the free nucleic acid base in aqueous solution. Gas phase experiments could be an alternative at this point. In particular, while gas phase studies of xanthine and xanthosine require an elaborate laser desorption setup, XMP${}^{-}$ could be brought into a molecular beam using an electrospray source.

The results obtained for the deactivation of XMP in aqueous solution are in good agreement with those obtained for the deactivation of methylated xanthine derivatives, where time scales of several 100 fs, ∼1 ps and 3.5–6.3 ps were found in water and acetonitrile [22]. Those values were attributed to relaxation dynamics via the five-membered ring, as well. Chen et al. [22] discussed the existence of long-lived optically dark states, such as ${}^{1}$n$\pi^{*}$, after excitation of methylated xanthine derivatives. Although we cannot strictly rule out the ${}^{1}$n$\pi^{*}$ state as a short-lived intermediate, we can exclude a substantial population of a long-lived dark ${}^{1}$n$\pi^{*}$ state or triplet states on the grounds of the observed GSR results.

### 3.2. XMP${}^{-}$

The excited-state deactivation of XMP${}^{-}$ in phosphate buffered H${}_{2}$O at pH 7 is governed by lifetime constants of $\tau_{1}$∼0.3 ps and $\tau_{2}$∼0.9 ps, comparable to the values for XMP. Again, $\tau_{1}$ is ascribed to fast motion away from the FC window and $\tau_{2}$ to the radiationless transition through the CI to the ground state. With $\tau_{\mathrm{GSR}}$∼4.4–5.9 ps, the ground state recovery time for XMP${}^{-}$ appears to be slightly longer than for XMP. As for neutral XMP, excitation to the second ${}^{1}\pi \pi^{*}$ state compared to the first ${}^{1}\pi \pi^{*}$ state seems to cause little changes of the observable dynamics, suggesting a rapid internal conversion from the upper to the lower ${}^{1}\pi \pi^{*}$ state on a time scale faster than our experimental resolution. Somewhat surprisingly, however, as noted in Section 2.2.2 above, it was not possible to obtain satisfactory fits to the fluorescence decay curves for XMP${}^{-}$ using two exponentials of varying amplitudes, but common lifetimes at the different emission wavelengths. Instead, a gradual increase of the fluorescence lifetime with increasing emission wavelength was evident. This observation, made after excitation mainly to the second ${}^{1}\pi \pi^{*}$ excited state, may hint at a sizeable gradient of the PEHS of the emitting state of XMP${}^{-}$. The motion of the excited wave packet following the potential energy gradient should lead to a rapid temporal red-shift of the fluorescence. Unfortunately, the amplitudes of our up-converted fluorescence signals cannot be quantitatively compared for experimental reasons; time-resolved measurements of the entire fluorescence spectra as possible by Kerr-gating would be needed here. We note, however, that the static fluorescence spectrum of XMP${}^{-}$ is blue-shifted despite its red-shifted absorption spectrum in comparison to XMP (Figure 2). In some not yet well understood way, this hints at significant differences between the excited potential energy hypersurfaces of XMP${}^{-}$ and XMP.

Since the results of the time-resolved experiments for XMP${}^{-}$ are similar to those for XMP, the relaxation mechanism can be expected to be similar, as well. This is not unlikely because deprotonation takes place at the N(3) position in the six-membered ring, where, as already noted in the Introduction, it might not have a large impact on the electronic structure of the xanthine moiety [9,13]. Interestingly, drastically different behaviours upon deprotonation were observed for the electronic deactivation dynamics of hypoxanthine and its nucleoside inosine [22,27,28,29]. Both molecules are deactivated via involvement of the out-of-plane deformation of the six-membered ring. However, deprotonation of inosine, which takes place at the six-membered ring, causes little difference in the excited-state dynamics [29]. Hypoxanthine, on the other hand, is deprotonated at the five-membered ring and shows an almost 20-fold increase of the fluorescence lifetime. The striking change has been explained by different electronic structures [29].

Besides these effects arising from deprotonation, a large impact on the electronic deactivation dynamics can also arise from protonation. For monoprotonated hypoxanthine and guanosine, for example, an increase of the excited-state lifetimes was found and attributed to the change of the potential energy topography compared to the neutral molecule [29,30]. Neutral hypoxanthine and its nucleoside inosine have been found to exhibit the fastest electronic deactivation rates of all purine bases studied to date. The observed fluorescence and ESA lifetimes of τ=0.18 and 0.21 ps, respectively, were assigned to the out-of-plane puckering deactivation channel involving the six-membered ring similar to guanine, but different from xanthine [28].

## 4. Materials and Methods

$5^{\prime }$-Xanthosine monophosphate was purchased as disodium salt from Sequoia Research Products and used as received. The sample solutions were prepared in acetate buffer at pH 3.6 (XMP) and in phosphate buffer at pH 7 (XMP${}^{-}$). Concentrations of 0.5–1 mM and 10 mM were used for the fluorescence up-conversion and transient absorption measurements, respectively. The relatively high concentrations employed in the transient absorption experiments were required due to the small amplitudes and short lifetimes of the observed ESA signals. Nevertheless, a formation of stacked XMP dimers is unlikely by the water hydration shell surrounding the molecules and the negative charges on the phosphate groups and the XMP${}^{-}$, while hydrogen-bonded dimers can be ruled out in the still highly dilute aqueous solutions due to the high excess of water. The acetate and phosphate buffers were prepared with double-distilled water (Carl Roth) using sodium acetate, acetic acid, Na${}_{2}$HPO${}_{4}$, NaH${}_{2}$PO${}_{4}$ and NaCl (all Sigma-Aldrich) in BioUltra quality. The sodium chloride was added to the phosphate buffer to adjust the salt concentration to the physiological level. The purities and concentrations of all solutions were checked before and after each time-resolved experiment by UV-Vis absorption spectroscopy on a Shimadzu UV-2401 spectrometer and fluorescence spectroscopy on a Horiba Jobin-Yvon Fluoromax-4 spectrometer.

The time-resolved fluorescence up-conversion and transient absorption experiments in our laboratory have been described in some detail elsewhere [28,31,32]. Both experiments were built around a regeneratively amplified 1-kHz Ti:Sa laser system (Clark MXR CPA 2001) providing pulses of ∼150 fs (FWHM) duration with 1000 μJ of energy at λ=775 nm. The 500 μJ of the laser output were used for the transient absorption setup, 400 μJ for fluorescence up-conversion. The pump pulses for both experiments were generated in home-built non-collinear optical parametric amplifiers (NOPAs) with subsequent frequency doubling stages and focused into the respective sample flow cells.

In the up-conversion experiment, the fluorescence was collected with a pair of off-axis parabolic mirrors and focused into a BBO crystal for type I non-collinear sum frequency generation (SFG). Residual pump light after the sample cell was blocked with a beam stop and a WG320 or a WG295 (Schott) filter, depending on the detection wavelength. The required time-delayed gate pulses were delivered by the Ti:Sa laser fundamental. The resulting SFG signal was detected at selected wavelengths after passing through a Jobin-Yvon HR-10 double monochromator by a photomultiplier (Hamamatsu R1527 P) connected to a preamplifier (Stanford Research SR-445) and computer-controlled single-photon counter (Stanford Research SR-400).

The broadband probe pulses for the transient absorption experiment were generated via supercontinuum generation in CaF${}_{2}$, yielding a usable spectrum between 330 nm $\leq \lambda_{\mathrm{probe}}\leq 700$ nm. Additional single-colour probe pulses in the UV range were generated in a second frequency-doubled NOPA. Probe and reference pulses for the broadband and single-colour detection were obtained by using the front and back reflections from a planar glass plate. The pump, probe and reference beams were focused into the sample cell, where the pump pulses were spatially and temporally overlapped with both probe pulses, and the reference pulses passed unexcited sample spots next to the pump pulses. The transmitted broadband probe and reference pulses were spectrally dispersed in a prism spectrograph and detected on a set of full frame transfer (FFT) back-thinned CCD cameras (S7030-0906, Hamamatsu and Entwicklungsbüro Stresing, Berlin, Germany). The single-colour UV pulses were recorded with two matched slow photodiodes (S1227-66BQ, Hamamatsu and Entwicklungsbüro Stresing, Berlin). Both CCD cameras and the photodiodes were read out simultaneously after each laser shot using home-written LabVIEW software.

All time-resolved measurements were performed in flow cells to exchange the sample volume between consecutive laser shots. The pump pulse energies were reduced to ≤120 nJ and ≤200 nJ for the fluorescence and absorption measurements, respectively. The optical pathlength in the time-resolved absorption measurements was d=0.1 mm to keep unwanted signals from the solvent, such as cross-phase modulation (XPM) and the long-lived absorption of solvated electrons (SEA) generated by multi-photon absorption, as low as possible [33]. The pure solvent was measured directly after each sample measurement to allow for an accurate correction for those artefacts. The optical pathlength in the fluorescence up-conversion measurements was d=1 mm. Each fluorescence measurement was repeated at least twice and each transient absorption scan three times to ensure reproducibility and reliability. The experimental time resolutions were on the order of Δt=150 fs and Δt=40 fs for the up-conversion and absorption data, respectively.

The experimental fluorescence decay curves provided by the up-conversion measurements were analysed using a simultaneous nonlinear least-squares fitting routine based on the Levenberg–Marquardt algorithm implemented in MATHEMATICA [34]. Each profile was described by a sum of exponentials starting at time zero convoluted with the instrument response function (IRF). The broadband transient absorption data were initially analysed in the same way in a preliminary fashion by simultaneous fitting at ten selected probe wavelengths, followed by a final global analysis by singular value decomposition (SVD) of the complete spectro-temporal transient absorption matrices [23,24,25] using a MATLAB [35] script. As described in detail in the literature, the SVD method provides a bias-free set of global time constants ($\tau_{i}$) and corresponding decay time-associated difference spectra (DADS${}_{i}$) describing the temporal and spectral evolutions of the recorded changes in optical density as a function of pump-probe delay time and probe wavelength using a minimal number of parameters. The time profiles measured in the deep-UV were again modelled with a sum of exponentials starting at time zero. The different methods gave excellent agreement (see the Results for details).

## 5. Conclusions

In conclusion, we have investigated the photophysics of xanthosine monophosphate following excitation to the first ${}^{1}\pi \pi^{*}$ and the second ${}^{1}\pi \pi^{*}$ electronic states in the neutral state and in the deprotonated state of the xanthine moiety by means of femtosecond time-resolved fluorescence up-conversion and transient absorption spectroscopy. Both neutral XMP and deprotonated XMP${}^{-}$ were found to exhibit similar dynamics, regardless of the initially excited ${}^{1}\pi \pi^{*}$ state. Observed ultrafast sub-picosecond processes in fluorescence, stimulated emission and absorption were attributed to rapid departures of the photo-excited wave packets from the initially populated Franck–Condon region. The short lifetimes of the excited-state absorption signals of *τ*∼1 ps, depending weakly on the deprotonation condition and the pump wavelength, show that the electronic relaxation through the conical intersection connecting the first ${}^{1}\pi \pi^{*}$ and ground states proceeds via barrierless, direct pathways. The assumed deactivation mechanism via out-of-plane deformation of the five-membered ring was theoretically predicted by Yamazaki et al. [15]. The perhaps slightly longer excited-state lifetime for XMP after excitation to the second ${}^{1}\pi \pi^{*}$ state (∼1.2 ps) compared to the first ${}^{1}\pi \pi^{*}$ state (∼0.8 ps) and compared to XMP${}^{-}$ hints at only very subtle differences of the potential energy hypersurfaces and the radiationless relaxation pathways. More detailed ab initio excited-state calculations on both XMP and XMP${}^{-}$ might be helpful to rationalize the dynamics.

## Figures and Tables

**Table 1:** Absorption maxima and extinction coefficients for XMP and XMP${}^{-}$.

Absorption Band	XMP	XMP${}^{-}$
$\lambda_{\max1}$/(nm)	263	277
$\epsilon_{\max1}$/(L·mol${}^{-1}$·cm${}^{-1}$)	8620	8140
$\lambda_{\max2}$/(nm)	235	249
$\epsilon_{\max2}$/(L·mol${}^{-1}$·cm${}^{-1}$)	8070	9530

**Table 2:** Time constants describing the electronic deactivation of XMP after photoexcitation at $\lambda_{\mathrm{pump}}=260$ and 243 nm provided by the global SVD analyses (〈av.〉 indicates the averaged values).

$\lambda_{\mathrm{pump}}$/nm	$\tau_{1}$/ps	$\tau_{2}$/ps	$\tau_{3}$/ps
260	0.39 ± 0.10	0.79 ± 0.13	4.0 ± 1.0
243	0.15 ± 0.03	1.19 ± 0.08	4.4 ± 1.0
〈 av. 〉	0.27±0.15	1.00±0.20	4.2±1.2

**Table 3:** Time constant values describing the deactivation of XMP${}^{-}$ after electronic excitation at $\lambda_{\mathrm{pump}}=278$, 260 and 243 nm from the global SVD analyses of the spectro-temporal absorption maps (〈av.〉 indicates the averaged values).

$\lambda_{\mathrm{pump}}$/nm	$\tau_{1}$/ps	$\tau_{2}$/ps	$\tau_{3}$/ps
278	0.21 ± 0.03	0.95 ± 0.04	–
260	0.41 ± 0.07	0.83 ± 0.08	4.9 ± 1.5
243	0.28 ± 0.03	0.87 ± 0.06	3.8 ± 0.8
〈 av. 〉	0.30 ± 0.11	0.88 ± 0.08	4.3 ± 1.0

## Appendix A

**Table A1:** Decay times $\tau_{i}$ and relative amplitudes $A_{i}$ from the analysis of the fluorescence up-conversion measurements for XMP after excitation at $\lambda_{\mathrm{pump}}=260$ nm (2σ error limits of last digits in parentheses).

$\lambda_{\mathrm{fluo}}/$nm	$\tau_{1,\mathrm{fluo}}/$ps	$A_{1,\mathrm{fluo}}$	$\tau_{2,\mathrm{fluo}}/$ps	$A_{2,\mathrm{fluo}}$	$\tau_{3,\mathrm{fluo}}/$ps	$A_{3,\mathrm{fluo}}$
290	0.28 (1)	0.94 (6)	0.91 (1)	0.06 (3)	−	−
330	0.28 (1)	0.82 (2)	0.91 (1)	0.18 (1)	−	−
350	0.28 (1)	0.74 (2)	0.91 (1)	0.26 (1)	−	−
460	−	−	0.91 (1)	0.96 (1)	36 (8)	0.04 (4)
500	−	−	0.91 (1)	0.97 (1)	36 (8)	0.03 (5)
560	−	−	0.91 (1)	0.96 (8)	36 (8)	0.04 (3)

**Table A2:** Decay times $\tau_{i}$ and associated amplitudes $A_{i}$ from the preliminary simultaneous analysis at selected probe wavelengths of the observed changes in optical density in the transient absorption measurements for XMP after excitation at $\lambda_{\mathrm{pump}}=260$ and 243 nm (2σ error limits of last digits in parentheses).

$\lambda_{\mathrm{pump}}/$nm	$\lambda_{\mathrm{probe}}/$nm	$\tau_{1}/$ps	$A_{1}/10^{-3}$	$\tau_{2}/$ps	$A_{2}/10^{-3}$
260	325	0.30 (20)	−0.8 (08)	0.9 (4)	1.7 (9)
	330	0.30 (20)	−1.0 (10)	0.9 (4)	2.0 (1)
	335	0.30 (20)	−1.3 (12)	0.9 (4)	2.1 (12)
	400	0.10 (4)	0.2 (2)	1.2 (2)	1.1 (2)
	420	0.10 (4)	1.3 (6)	1.2 (2)	0.8 (2)
	425	0.10 (4)	1.4 (6)	1.2 (2)	0.7 (2)
	460	0.10 (4)	0.9 (6)	1.2 (2)	0.2 (2)
	560	0.18 (4)	0.9 (5)	1.2 (2)	0.04 (2)
	600	0.18 (4)	1.0 (5)	−	−
	640	0.18 (4)	1.6 (4)	−	−
	244	0.60 (40)	7 (07)	0.9 (4)	−8 (8)
243	325	0.30 (20)	−0.6 (6)	1.2 (2)	1.8 (4)
	330	0.30 (20)	−0.8 (6)	1.2 (2)	2.1 (5)
	335	0.30 (20)	−1.1 (6)	1.2 (2)	2.4 (6)
	400	0.10 (4)	1.0 (6)	1.2 (2)	1.4 (2)
	420	0.10 (4)	1.8 (6)	1.2 (2)	1.3 (2)
	425	0.10 (4)	2.0 (6)	1.2 (2)	1.1 (2)
	460	0.10 (4)	1.8 (6)	1.2 (2)	0.5 (2)
	560	0.18 (4)	1.3 (5)	1.2 (2)	0.2 (2)
	600	0.18 (4)	2.3 (5)	1.2 (2)	0.1 (1)
	640	0.18 (4)	1.9 (4)	−	−
	244	0.4 (3)	3. (02)	1.1 (2)	−1.8 (4)

**Table A3:** Decay times $\tau_{i}$ and relative amplitudes $A_{i}$ from the analysis of the fluorescence up-conversion measurements for XMP${}^{-}$ after excitation at $\lambda_{\mathrm{pump}}=260$ nm (2σ error limits of last digits in parentheses).

$\lambda_{\mathrm{fluo}}/$nm	$\tau_{1,\mathrm{fluo}}/$ps	$A_{1,\mathrm{fluo}}$	$\tau_{2,\mathrm{fluo}}/$ps	$A_{2,\mathrm{fluo}}$
330	0.51 (1)	0.82 (2)	1.7 (5)	0.11 (6)
350	0.73 (2)	0.74 (2)	4 (1)	0.03 (1)
460	0.86 (4)	−	4 (1)	0.06 (3)
480	1.0 (2)	−	4(1)	0.03 (3)

**Table A4:** Decay times $\tau_{i}$ and associated amplitudes $A_{i}$ from the preliminary simultaneous analysis at selected probe wavelengths of the observed changes in optical density in the transient absorption measurements for XMP${}^{-}$ after excitation at $\lambda_{\mathrm{pump}}=260$, 260 and 243 nm (2σ error limits of last digits in parentheses).

$\lambda_{\mathrm{pump}}/$nm	$\lambda_{\mathrm{probe}}/$nm	$\tau_{1}/$ps	$A_{1}/10^{-3}$	$\tau_{2}/$ps	$A_{2}/10^{-3}$	$\tau_{3}/$ps	$A_{3}/10^{-3}$
278	325	0.28 (3)	−0.4 (4)	0.97 (5)	0.8 (2)	−	−
	330	0.28 (3)	−0.5 (4)	0.97 (5)	1.0 (2)	−	−
	335	0.28 (3)	−0.6 (4)	0.97 (5)	1.1 (2)	−	−
	400	0.28 (3)	−0.4 (4)	0.97 (5)	1.3 (2)	−	−
	420	−	−	0.97 (5)	1.4 (1)	−	−
	425	−	−	0.97 (5)	1.4 (1)	−	−
	460	0.28 (3)	0.1 (1)	0.97 (5)	1.0 (2)	−	−
	560	0.28 (3)	0.5 (3)	0.97 (5)	0.6 (2)	−	−
	600	0.28 (3)	0.6 (3)	0.97 (5)	0.4 (2)	−	−
	640	0.28 (3)	0.9 (3)	0.97 (5)	0.2 (2)	−	−
	243	0.8 (2)	1.7 (17)	1.3 (3)	−2.5 (11)	−	−
260	325	0.28 (3)	−1.8 (5)	0.97 (5)	3.2 (4)	4	0.3 (2)
	330	0.28 (3)	−2.0 (5)	0.97 (5)	3.8 (4)	4	0.3 (2)
	335	0.28 (3)	−2.0 (5)	0.97 (5)	4.0 (4)	4	0.4 (2)
	400	0.28 (3)	−0.6 (5)	0.97 (5)	2.4 (4)	4	0.2 (2)
	420	−	−	0.97 (5)	2.4 (2)	4	0.2 (1)
	425	−	−	0.97 (5)	2.3 (2)	4	0.2 (1)
	460	0.28 (3)	0.1 (1)	0.97 (5)	1.7 (2)	−	−
	560	0.28 (3)	0.8 (4)	0.97 (5)	1.0 (2)	−	−
	600	0.28 (3)	1.4 (4)	0.97 (5)	0.5 (2)	−	−
	640	0.28 (3)	2.0 (2)	−	−	−	−
	243	0.8 (2)	6.2 (62)	1.3 (3)	−8.3 (70)	4	−0.4 (2)
243	325	0.28 (3)	−2.4 (6)	0.97 (5)	3.4 (4)	4	0.4 (2)
	330	0.28 (3)	−3.2 (6)	0.97 (5)	4.4 (5)	4	0.5 (2)
	335	0.28 (3)	−3.5 (6)	0.97 (5)	5.0 (5)	4	0.6 (2)
	400	0.28 (3)	−0.7 (6)	0.97 (5)	2.8 (4)	4	0.4 (2)
	420	−	−	0.97 (5)	2.7 (2)	4	0.4 (1)
	425	−	−	0.97 (5)	2.6 (2)	4	0.4 (1)
	460	−	−	0.97 (5)	2.0 (2)	4	0.2 (1)
	560	0.28 (3)	1.0 (4)	0.97 (5)	1.2 (2)	−	−
	600	0.28 (3)	2.1 (4)	0.97 (5)	1.0 (2)	−	−
	640	0.28 (3)	2.3 (4)	0.97 (5)	0.3 (2)	−	−
	243	0.8 (2)	2.2 (22)	1.3 (3)	−2.8 (24)	4	−0.3 (1)
